# 537 Laser-Assisted Drug Delivery in the Treatment of Hypertrophic and Keloid Scars: A Systematic Review

**DOI:** 10.1093/jbcr/irac012.166

**Published:** 2022-03-23

**Authors:** Rendell Bernabe, Tiffany Calero, Joshua Lin, Justin Dang, Paloma Madigral, Haig A Yenikomshian, Justin Gillenwater

**Affiliations:** LAC + University of Southern California, Los Angeles, California; University of Southern California, Los Angeles, California; Keck School of Medicine, University of Southern California, Los Angeles, California; Sidney Kimmel Medical College at Thomas Jefferson University, Costa Mesa, California; University of Southern California, Los Angeles, California; USC/LAC+USC Medical Center, Los Angeles, California; USC/LAC+USC Medical Center, Los Angeles, California

## Abstract

**Introduction:**

Hypertrophic scars (HTS) and keloids (K) cause significant morbidity and disfigurement. Care of HTS and keloids range from less invasive treatments, such as pressure garments and silicone products, to more invasive treatments, such as intralesional injections (ISI) of medication, and surgical excision. Laser-Assisted Drug Delivery (LADD) is becoming a more popular treatment for HTS and K. The ablative fractional laser creates microchannels in the skins which allows the delivery of drugs into deeper skin layers. We have conducted a systematic review of the literature to assess the effectiveness of LADD in the treatment of HTS and K.

**Methods:**

A search was performed on PubMed between January 1998-August 2021 using the following keywords: Laser Assisted Drug Delivery, Laser Combined Drug Delivery, Laser Drug Delivery, Laser Drug, Hypertrophic, Burn, Keloid, Scar. Inclusion criteria was the use of LADD in HTS or K. Exclusion criteria were studies in animal models, case reports, reviews, and non-english articles. One article was excluded due to concerns of plagiarism.

**Results:**

A total of 11 articles were included in the final review. One study found no difference in HTS outcomes between LADD of corticosteroids (CTS) and laser with topical petrolatum used as a control. Two prospective studies found that LADD of CTS led to improved HTS outcomes. Two split-scar studies found no difference in K outcomes between LADD of CTS and ISI of CTS. One prospective study assessed LADD of CTS in K and found statistically significant improvements in scar outcomes. A retrospective study looking at K outcomes found a 50% mean improvement when treated with LADD of CTS. A split-scar study found statistically significant better HTS outcomes with LADD of 5-FU compared to topical 5-FU alone. One study found no difference in HTS outcomes between LADD of CTS and 5-Fluorouracil (5-FU). Two studies assessed both HTS and K. The first study found that both LADD of 5-FU and LADD of CTS led to significantly better scar outcomes when compared to laser monotherapy. The other reported that HTS outcomes were significantly better with LADD of Botulinum toxin A and K outcomes were better with ISI of Botulinum Toxin A.

**Conclusions:**

The use of LADD is effective in the setting of HTS. There is evidence that LADD of scar modulating agents to HTS lesions is more effective than topical delivery or ISI of the same agent. LADD shows promise as an alternative treatment for K as it displays similar efficacy as ISI.

